# Multiple high-risk fertility behaviours and children under five mortality survivors among ever-married women of reproductive age in Nigeria

**DOI:** 10.1186/s13690-023-01192-2

**Published:** 2023-09-27

**Authors:** Obasanjo Afolabi Bolarinwa, Julia Marie Hajjar, Oluwatobi Abel Alawode, Kobi V. Ajayi, Adedoyin Tinuoya Roberts, Sanni Yaya

**Affiliations:** 1 Department of Public Health, York St. John University, London, UK; 2 Department of Demography and Population Studies, University of Witwatersrand, Johannesburg, South Africa; 3 Interdisciplinary School of Health Sciences, Faculty of Health Sciences, University of Ottawa, Ottawa, ON Canada; 4 Department of Sociology and Criminology & Law, University of Florida, Gainesville, FL 32611 USA; 5 Department of Health Behavior, School of Public Health, Texas A&M University, College Station, TX USA; 6 Department of Social Research Methods, University of Brighton, Brighton, UK; 7 School of International Development and Global Studies, Faculty of Social Sciences, University of Ottawa, Ottawa, ON Canada; 8 The George Institute for Global Health, Imperial College London, London, UK

**Keywords:** Under-five mortality, High-risk fertility behaviours, Ever-married women of reproductive age, Global health, DHS, Nigeria

## Abstract

**Background:**

Multiple high-risk fertility behaviours (MHRFBs), including maternal age < 18 or > 34 years old, a birth order 4+, and birth spacing < 24 months, can directly or indirectly affect survival outcomes among under-five children. There is a dearth of available information and data about these two phenomena in Nigeria. Thus, this study evaluates the prevalence of MHRFBs and examines the association between MHRFBs and under-five mortality survival (U5M) outcomes among ever-married women of reproductive age in Nigeria.

**Methods:**

This study used the recent secondary datasets from the Nigerian Demographic Health Surveys conducted in 2018, with a total sample size of 10,304 women of reproductive age. The outcome variable was MHRFBs. Multivariable logistic regression analysis was employed to examine the association between U5M and MHRFBs. Odds ratios with a p-value of less than 0.05 were considered significant.

**Results:**

It was found that among women who had MHRFBs, U5M was prevalent, particularly in young maternal age (< 18 years) and within short birth intervals (< 24 months). The adjusted odds ratio of the association between MHRFBs and U5M shows the experience of MHRFBs, in addition to other factors such as household wealth index, type of marriage, and sex of child, to be significant predictors for U5M. The odds were higher for U5M to occur among women who had experienced MHRFBs compared to those who have not had an experience of MHRFBs [aOR = 1.48; 95%CI: 1.02–2.17 ]. Similarly, the odds of U5M occurrence among women in polygamous marriages are higher compared to those in monogamous unions [aOR = 1.35; 95% CI: 1.10–1.65]. While under-five children born in the richest households (wealth quintiles) are less likely to die compared to those born in the poorest households [aOR = 0.64; 95% CI: 0.41–1.01].

**Conclusion:**

This study concludes that women in Nigeria who engaged in MHRFBs, particularly maternal ages < 18 years and short birth intervals (< 24 months), were more likely to experience U5M. Furthermore, children born to women who received post-natal care after delivery were more likely to survive U5M, as were children born to women with educated partners. We recommend strengthening educational opportunities and creating adaptive reproductive health education programs for ever-married women of reproductive age in Nigeria.

## Background

High-risk fertility behaviors (HRFBs) include maternal age < 18 or > 34 years old, a birth order 4+, and birth spacing < 24 months [1, 2]. Multiple High-risk fertility behaviors (MHRFBs) involve two or more of these [1–3]. MHRFBs occur in part because women are unable to access or fulfil the need to use and/or plan contraception due to disruption or complete partner disapproval, disempowerment, or lack of negotiating power in decision-making around contraceptive adoption, which often results in unintended or mistimed pregnancies [3–5]. MHRFBs pose a significant threat to the health of both mother and child during pregnancy and the postpartum period, thereby contributing to the continued high rates of maternal, neonatal, and child under-five morbidity and mortality globally and in Nigeria [1, 6, 7].

Studies have shown that fertility and infant mortality, particularly under-five mortality (U5M), are closely related by several factors; for instance, MHRFBs are reported to be risk factors for U5M [8–10]. Nigeria is a country with extraordinarily high rates of maternal and child mortality: over 25% of maternal, newborn, and child deaths in sub-Saharan Africa (SSA) occur in the country [11]. In 2019, the country had one of the five highest U5M rates globally [8], accounting for nearly one-third of all U5M [4, 7]. One recent study reported that the rate of U5M was 132 children per 1000 live births in 2018 [10], a staggering number compared to 4.7 children per 1000 live births in Canada that same year [9] and higher compared to rates in other African nations [11]. Several pervasive gaps and challenges continue to drive the high rates of maternal, neonatal, and child under-five morbidity and mortality rates in Nigeria.

Despite the various government-led and third-sector-supported interventions in place to curb this alarming rate, there still exists an uneven gap across subnational levels, which are directly and/or indirectly able to sabotage efforts in place. According to the most recent Nigeria demographic health survey report, not only are U5M rates high, but the total fertility rate of 5.3 children per woman (state levels of 7.3 versus 3.4) is also very high [8], particularly as the short birth intervals and early and/or advanced maternal ages of women involved in multiple births continues to put lives of women and children at high of risk of death and other adverse health outcomes. While evidence from recent studies in Nigeria shows that U5M and the number of children born decline with mothers’ education and increasing household wealth, there continue to be regional disparities in the occurrence of U5M, with the northern region accounting for the highest share of U5M [8, 9].

In light of the adverse implications of U5M and MHRFBs on reproductive health and fertility, it is worthwhile to investigate these two phenomena and their cross-cutting impact on maternal and child health outcomes in Nigeria for numerous reasons. The consequences of high fertility have been well documented, ranging from increased child and maternal morbidity, poor or complete lack of education for large families, negative economic growth, and environmental threats [12]. Furthermore, while almost the entirety of preventable maternal deaths occurred in low- and middle-income countries (LMICs), Nigeria accounts for 20% of global maternal deaths and stands as the country with the highest child under five deaths (844 deaths per 1000 live births) [13, 14], and underperforms across several maternal, neonatal, and infant health indices. These statistics signal an urgent need to address confounding factors contributing to the country’s excess maternal, infant, and child morbidity and mortality burden.

Other studies have only examined these two phenomena bounded within a particular region or setting, studied the impact on health outcomes, were not specific to Nigeria, and/or utilised nonrepresentative data [5, 15, 16], thus limiting the generalizability of these findings. Since U5M and MHRFBs are major health issues in Nigeria, a more expanded and rigorous study using nationally representative data is needed to test the association between the two phenomena.

As efforts to combat U5M reverberate and calls to reduce U5M and MHRFBs in Nigeria abound, understanding the cumulative determinants and nuances associated with U5M and MHRFBs is critical. This study uses data from the 2018 Nigerian Demographic and Health Survey to specifically evaluate the prevalence of MHRFBs and examine the association between MHRFBs and U5M survivor among ever-married women of reproductive in Nigeria. Results from this study have serious implications for global, national, and regional efforts to strengthen policies, practices, and research. Findings would thereby inform tailored and localised strategies and programs to protect and promote women’s reproductive health and overall well-being.

## Data & Methods

### Study design and participants

This cross-sectional study utilised secondary data from the 2018 Nigerian Demographic and Health Survey (NDHS), specifically the individual recode data file. The Demographic Health Surveys (DHSs) are conducted in over 85 LMICs, and are well known for its nationally representative sampling design. The National Population Commission conducted the survey with technical and financial assistance from ICF Macro and USAID [17]. The survey covered all territories in Nigeria, including rural and urban areas, all 36 states, the Federal Capital Territory, Abuja, and all geo-political zones, namely South East, South-South, South West, North East, North West and North Central. Questions regarding participants’ socio-demographic characteristics, maternal and child health, and other sexual and reproductive health-related indicators such as sexual intimate partner violence, family planning use, abortion, HIV testing, etc., are usually asked among women aged 15–49 using a structured questionnaire [18].

**Fig. 1:**
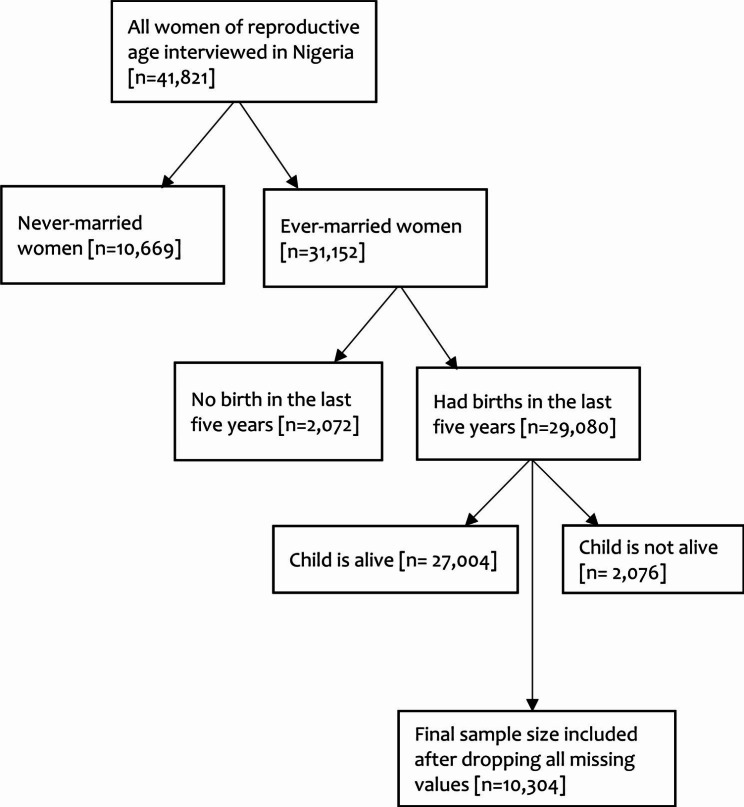
Flow chart of study respondents selection

The sampling design for the DHS involves a two-stage sampling procedure that consists of primary survey units (PSUs) from which participants are randomly selected from clusters. Specifically, the sampling is generally representative at the national, residential (urban-rural), and regional (state) levels. In the stratified two-stage cluster sampling design, the first stage included enumeration areas (EAs), which were drawn from census files of the country (i.e. 2006 census in Nigeria), and the second stage involved the selection of households from a list of households that were drawn from each of the EAs selected in the first stage [17]. Nigeria comprises 37 states, including the Federal Capital Territory (FCT)-Abuja. These states were divided into urban and rural sectors, resulting in 74 distinct sampling categories. From these, 1,400 EAs were chosen based on their size. In each EA, 30 households were systematically selected with equal probability, and 41,821 women between the ages of 15 to 49 from 40,427 households were selected as a representative sample of the country. More information on the DHS methodology has been published elsewhere [19]. This present study focused on 10,304 ever-married women of reproductive age 15–49 years interviewed in the 2018 survey in Nigeria who met the criteria for MHRFBs and had at least one childbirth within the last five years preceding the NDHS survey and provided information about the reproductive and maternal healthcare used during last pregnancy. The eligible respondents selection process is shown in figure 1 below. All women who had never been married or were childless were excluded from this study [20]. The DHS datasets used in this study are publicly available on the DHS website and can be downloaded for free upon request via https://dhsprogram.com/data/available-datasets.cfm.

### Study variables

#### Response variable

The response variable in the study is the survival of under-five children, which was collected by verbal declaration or report by the mothers of these children. The survival status of under-five children in Nigeria was coded as (alive = 0; dead = 1). The survival time (months) was derived for each child aged 0–59 months using survival status at the 59th month, age at death, and interview date. Children who were not reported to have experienced death during the survey were coded 0; otherwise, 1 in the analysis [21].

#### Main explanatory variable

The key explanatory variable in this study is MHRFBs. Similar to Das et al. [20], we conceptualised MHRFBs based on the following criteria: (1) women whose age at first birth was < 18 years, 2) latest child of order three or higher, and 3) birth spacing < 24 months (i.e., the most recent child born was within 24 months of the prior birth). The response of Yes or No to each of these questions in the survey was used to generate the outcome variable in this study. MHRFBs were coded Yes (1) if a woman responded Yes to at least two of the above criteria and No (0) otherwise [20].

#### Other explanatory variables

This study also included other explanatory variables based on the literature on child survival in Nigeria and similar contexts [6, 7]. These include the age of the women, age at first marriage, highest level of education, partner’s highest level of education, type of place of residence, type of marriage, work status, household wealth index, level of mass media exposure, sex of the head of the household, religion, number of antenatal care visits during pregnancy and postnatal care check for newborns. [22–24].

### Statistical analyses

Data analyses in this study were implemented in three steps using the STATA 17.0 version (StataCorp College Station, TX, USA). Survey weight was generated and applied to the dataset and every step of the data analysis to adjust for differences in population sizes of each region in Nigeria. Weighting the data will extrapolate the analysis output to other areas not covered during the survey in order to enhance the generalizability of the study’s findings [22, 24]. Descriptive statistics of all study variables were presented at the univariate level. In the bivariate analysis, the Chi-square test of association was employed to see the categorical differences and test the association between MHRFBs and other covariates and under-five mortality. A multivariate logistic regression was employed with a total of three models fitted, as presented in Table 1. “Model 1” accounted for the unadjusted model (cOR) of the relationship between the MHRFBs and U5M. “Model 2” presents the adjusted model (aOR) of the relationship between the MHRFBs and under-five mortality, accounting for the effect of other explanatory variables. The models were built based on what has been done in similar studies on the determinants of under-five mortality in Nigeria [25, 26]. These models were presented with their corresponding confidence intervals (CIs), and statistical significance was set at p < 0.001 and p < 0.005.

**Table 1:** Binary logistic regression models of the relationship between multiple high-risk fertility behaviors and under-five survival status among ever-married women of reproductive age in Nigeria

Under-five Mortality	Model 1 cOR [95% CI]	Model 2 aOR [95% CI]
Multiple High-Risk Fertility		
No	ref	
Yes	1.95 [1.35–2.82] ***	1.48 [1.02–2.17] **
**Highest Level of Education**		
No Education		ref
Primary		1.17 [0.90–1.51]
Secondary		1.15 [0.86–1.53]
Higher		1.34 [0.81–2.22]
**Household Wealth Index**		
Poorest		ref
Poorer		1.06 [0.81–1.39]
Middle		0.93 [0.68–1.28]
Richer		0.88 [0.62–1.26]
Richest		0.64 [[0.41–1.01] **
**Age at first Marriage**		
< 18		ref
18+		1.07 [0.86–1.33]
**Type of Marriage**		
Monogamy		ref
Polygamy		1.35 [1.10–1.65] ***
**Partner’s Highest Level of Education**		
No Education		ref
Primary		0.92 [0.68–1.25]
Secondary		0.73 [0.56–0.94] **
Higher		0.74 [0.51–1.07]
**Religion**		
Christianity		ref
Islam		1.11 [0.85–1.44]
Others		0.20 [0.03–1.21]
**Level of Exposure to Mass Media**		
No Exposure		ref
Low Exposure		1.14 [0.92–1.43]
High Exposure		1.12 [0.81–1.55]
**Sex of Child**		
Male		ref
Female		0.84 [0.70–1.01] **
**Type of Place of Residence**		
Urban		ref
Rural		0.91 [0.71–1.16]
**Sex of Household Head**		
Male		ref
Female		1.23 [0.89–1.70]
**Region**		
North Central		
North East		1.21 [0.86–1.69]
North West		1.23 [0.90–1.69]
South East		1.08 [0.73–1.61]
South South		1.08 [0.72–1.63]
South West		0.76 [0.51–1.13]
**Antenatal Care Visits**		
None		ref
1–3		0.91 [0.70–1.20]
4+		0.84 [0.67–1.06]
**Postnatal Care for Child**		
No		ref
Yes		0.59 [0.46–0.76] ***

### Ethical approval and consent to participate

Since the author of this manuscript did not collect the data, we sought permission from the MEASURE DHS website, and access to the data was provided after our intent for the request was assessed and approved on the 20th of January, 2022. Nigeria Population Council and related agencies in Nigeria ethically accept the DHS surveys. The interviewed women gave written and/or verbal consent during the surveys. Further information about ethics and respondents’ privacy can be found at the following link: https://dhsprogram.com/Methodology/Protecting-the-Privacy-of-DHS-Survey-Respondents.cfm.

## Results

**Fig. 2:**
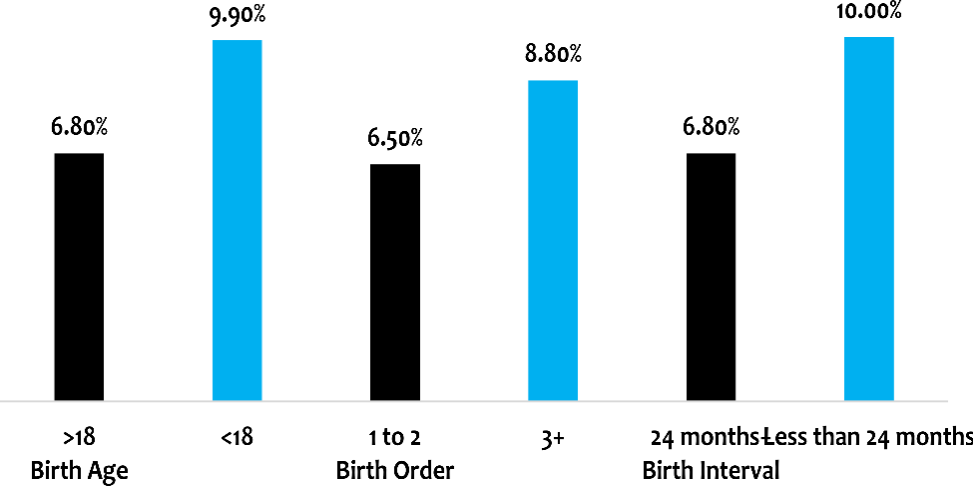
Prevalence of under-five mortality by multiple high-risk fertility behaviors among ever-married women of reproductive age in Nigeria

The distribution of under-five mortality by the different categories of high-risk fertility behaviors showed that the prevalence is higher among women who had a birth at less than 18 years (9.9%), had a birth order of three or more (3+) (8.8%) and a birth interval of fewer than 24 months (10%) as shown in figure 2 below.

Table 2 shows the weighted percentage distribution of infant survival status by MRHFB, socioeconomic status, demographic, and health services utilisation. The study found that 90% of the women in the study have experienced at least one indicator of multiple high-risk fertility. 49% of the women in the study are aged 25–34, while 97% are currently married. The analysis also showed that more than half of the women married at 18 years and above (51%), while 40% had no education, and 30% reported that their partners had no formal education. 58% of the respondents reside in rural areas, 29% live in the North West region of the country, while 87% of the women in the study are multiparous, and 71% are monogamous.

**Table 2:** Weighted percentage distribution of infant survival status by multiple high-risk fertility behaviors, socio-demographic variables and health services utilisation

	n [%]	Under-five mortality	p-value
**Multiple High-Risk Fertility**			0.001
No	1,020 [9.9]	5.14	
Yes	9,284 [90.1]	8.64	
**Age of Mother**			< 0.001
15–24	1,690 [16.4]	8.97	
25–34	4,992 [48.5]	6.68	
35+	3,622 [35.1]	9.67	
**Marital Status**			0.055
Married	9,968 [96.7]	8.24	
Cohabiting	335 [3.3]	5.45	
**Age at first Marriage**			< 0.001
< 18	5,067 [49.2]	9.61	
18+	5,237 [50.8]	6.71	
**Highest Level of Education**			< 0.001
No Education	4,106 [39.9]	10.23	
Primary	1,677 [16.3]	8.86	
Secondary	3,449 [33.5]	5.99	
Higher	1,072 [10.4]	5.85	
**Partner’s Highest Level of Education**			< 0.001
No Education	3,119 [30.3]	11.2	
Primary	1,643 16.0]	8.34	
Secondary	3,875 [37.6]	6.46	
Higher	1,666 [16.2]	6.19	
**Type of Place of Residence**			< 0.001
Urban	4,339 [42.1]	6.66	
Rural	5,964 [57.9]	9.01	
**Region**			< 0.001
North Central	1,587 [15.4]	7.66	
North East	1,696 [16.5]	9.51	
North West	2,987 [28.9]	11.62	
South East	1,103 [10.7]	6.1	
South South	997 [9.7]	5.27	
South West	1,933 [18.8]	5.03	
**Parity**			0.044
Primiparity	1,342 [13.0]	6.67	
Multiparity	8,960.72 [87.0]	8.34	
**Type of Marriage**			< 0.001
Monogamy	7,273 [70.6]	7.1	
Polygamy	3,030 [29.4]	10.68	
**Religion**			< 0.001
Christianity	4,519 [43.9]	6.37	
Islam	5,726 [55.6]	9.83	
Others	58 [0.6]	1.19	
**Level of Mass Media Exposure**			< 0.001
No Exposure	3,380 [32.8]	9.45	
Low Exposure	4,660 [45.2]	8.13	
High Exposure	2,263 [22.0]	5.97	
**Wealth Index**			< 0.001
Poorest	2,044 [19.8]	9.94	
Poorer	2,118 [20.6]	10.1	
Middle	2,123 [20.6]	8.13	
Richer	1,988 [19.3]	6.71	
Richest	2,029 [19.7]	4.98	
**Sex of Household Head**			**< 0.001**
Male	9,474 [91.9]	8.27	
Female	829 [8.1]	6.73	
**Antenatal Care during pregnancy**			< 0.001
None	2,118 [20.6]	10.79	
1–3	1,615 [15.7]	8.91	
4+	6,570 [63.8]	7.06	
**Sex of the child**			0.004
Male	5,316 [51.6]	8.89	
Female	4,987 [48.4]	7.34	
**Postnatal check for Child**			< 0.001
No	7,897 [76.7]	9.19	
Yes	2,406 [23.3]	4.7	

Model 1: unadjusted results of multiple high-risk fertility behaviours and children under five survivors

Model 2: Accounted for co-founders (education, wealth index, age at first marriage, type of marriage, partner’s education, religion, mass media exposure, sex of child, place of residence, sex of household head, region, antenatal care visits, postnatal care for child)

CI = Confidence Interval; cOR = Crude Odds Ratio; aOR = Adjusted Odds Ratio; ref = Reference; ***=p < 0.001; **=p < 0.005

Furthermore, 56% are Muslims, 44% are Christians, 45% have been found to have low exposure to mass media, and 21% of the respondents are from poorer and middle households. In addition, 8% of the women reported that females head their households, and 52% of the women reported the sex of the child as male. Finally, 64% of the women attended four or more antenatal visits during their last pregnancy, while 77% reported that their newborn had a postnatal check after birth.

Furthermore, the prevalence of U5M by MHRFBs and other background characteristics showed that the prevalence of U5M among women with experience of MHRFBs is 9%. The prevalence of U5M by maternal age shows the highest prevalence among mothers aged 35+ (10%); the prevalence is 8% for married women compared to 5% among cohabiting mothers. It was also found that the prevalence of U5M among these respondents is higher for women who married less than 18 years of age (10%). For the educational level of the women, U5M was found to be highest among women with no formal education (10%), and the prevalence was 11% for those whose partners had no formal education. U5M prevalence was 9% in rural areas compared to 7% among urban residents; the prevalence is highest in Northern geopolitical zones, with 12% in the North West region. Women with no mass media exposure have the highest prevalence of U5M (9%), and women in poorer households have the highest prevalence of U5M (10%). The analysis also shows that U5M is more common in male-headed households (8%). Women who had no ANC visits during pregnancy have an 11% U5M prevalence, while women who reported that their newborn did not have a postnatal check after birth have a 9% U5M prevalence.

Table 1 in Model 2 shows the adjusted results between MHRFBs, U5M survivors and selected covariates.The findings showed that under-five children born to women with experience of MHRFBs were 48% more likely to experience U5M compared to children of women with no experience MHRFBs [aOR = 1.48; 95% CI: 1.02–2.17]. Household wealth index was also statistically associated with U5M; those from the richest households are 36% less likely to die compared to those being born in the poorest household [aOR = 0.64; 95% CI: 0.41–1.01]. Under-five children born in polygamous marriages were 35% more likely to experience U5M compared to those born in monogamous marriages [aOR = 1.35; 95% CI: 1.10–1.65]. It was also found that under-five children of women whose partners have a secondary education were 27% less likely to die compared to those whose partners do not have formal education [aOR = 0.73; 95% CI: 0.56–0.94]. Female children were 16% less likely to die compared to males [aOR = 0.84; 95% CI: 0.70–1.01], and it was also found that children who had postnatal checks after birth were 41% less likely to experience U5M compared to those who were not checked after birth [aOR = 0.59; 95% CI: 0.46–0.76].

## Discussion

Several pervasive gaps and challenges continue to drive the high rates of maternal, neonatal, and child under-five morbidity and mortality rates in Nigeria. This study evaluates the prevalence of MHRFBs and examines the association between MHRFBs and U5M survivors among ever-married women of reproductive in Nigeria. In particular, the relationship between MHRFBs and U5M in Nigeria has not been adequately assessed. To address this gap, our study analysed secondary data from the 2018 NDHS to explore the association between MHRFBs and the survival status of under-five children in Nigeria. In addition, several covariates of interest were considered, including women’s age, age at first marriage, educational level, place of residence, type of marriage, work status, household wealth index, sex of household head, religion, number of antenatal care visits, and postnatal check for the newborn. The factors that were significantly associated with U5M in Nigerian children in our study included the maternal experience of MHRFBs, household wealth index, type of marriage, partner’s education level, sex of the child, and whether the child received postnatal care after birth. Being one of the most highly populated countries, both in SSA and globally, addressing broader systemic issues as well as identifying and addressing root causes of health inequities by considering the social determinants of health will be pivotal to ignite change within the country by reducing the exorbitant U5M rates and continuing to work towards meeting the SDG targets by 2030. However, it’s noteworthy that policymakers and public health stakeholders are informed that the interpretation of this study’s result should be implemented cautiously for policy and developmental decisions because the dataset was collected 5-years before these findings.

In our study, children of women who experienced MHRFBs were 48% more likely to experience U5M compared to children of women with no MHRFBs experience. Similarly, another Nigerian study reported that children of mothers with a higher birth order (2–4+) who had a shorter birth interval (< 24 months) experienced a higher risk of neonatal mortality [27]. Another study in Nigeria reported that the highest infant mortality rates occurred in newborns born to women who experienced at least two HRFBs and in male infants [28]. The literature also reports similar findings more broadly in SSA. An analysis of 205 DHS surveys from 57 developing countries between 1985 and 2013 reported that the countries that improved their modern contraceptive prevalence rates significantly reduced high-risk births attributed to MHRFBs [29]. These changes were largely attributed to improvements in family planning education and women’s education- addressing these factors reduced high-risk birth rates due to MHRFBs that included high parity, small birth spacing (< 24 months) and mature maternal age over 35 [30]. The literature also identified the importance of including men and community religious leaders in discussions around family planning [31]. Overall, the literature suggests that enhancing family planning and strengthening women’s education must address root causes in order to mitigate high-risk births attributed to MHRFBs [30, 32].In Ethiopia, women who experience MHRFBs are more likely to have a child who dies before the age of 5 [6].

Similarly, a large study that analysed DHS data from 32 countries in SSA, the Middle East, North Africa and South Asia from 1986 to 2017 revealed that women who experienced MHRFBs (particularly if women were under the age of 18 when they gave birth and had a birth interval of less than 24 months) put children at a higher risk for U5M [1]. Children of women who experienced at least two HRFBs in the study were 39% more likely to experience U5M [1]. This emphasises the impact of MHRFBs on child mortality rates, and mitigation strategies to address the root cause of MHRFBs are thus crucial to lowering the rates of U5M in Nigeria, as well as within the region of SSA.

Our study shows that children born to women in the richest household wealth index were 36% less likely to experience mortality compared to children of women in the poorest households. This aligns with previous studies, which have reported that children in the poorest wealth quintiles are at the highest risk of U5M, and risk decreases with increasing wealth quintile [33]. Poverty is a significant barrier to seeking appropriate, high-quality care for infants at legitimate health facilities: parents may wait or seek lower-cost alternative herbal medicines before seeking more costly care [34]. It is important to note that funding for child health services in Nigeria is quite inadequate, with limited funding and any existing funding stemming from donors or private sources [35]. Given that a large concentration of the poor resides in the country’s rural regions [36], poverty is a significant barrier in Nigeria and has incredibly impactful consequences.

This is especially concerning given the compounded challenges associated with seeking care for poorer individuals who already experience health disparities. Issues surrounding lack of transportation and far distance to healthcare facilities, paired with the high cost of medications, poor healthcare staffing, and a weak health system [37], make it even more difficult to access equitable healthcare in the country, particularly for poorer Nigerians.

Children of women in polygamous marriages were 35% more likely to experience U5M compared to children born into monogamous marriages was reported in this study. Almost 25% of marriages in SSA are polygamous, and almost 25% of children in SSA are born into polygamous marriages [38]. Interestingly, research in Sierra Leone reports that the higher fertility levels observed in women in polygamous marriages may be due to competition between female partners, with each woman in the relationship wanting to have the most children to ensure greater financial security [39]. This was also reported by Ndiaye et al. [40] in their study on high-parity women of advanced maternal age in Niger and Togo. The study indicated that women in polygamous marriages may want more children to ensure greater financial and relationship security [40]. Polygamy can be beneficial or harmful to a child’s health and mortality: some studies report a protective effect on child mortality due to having more co-parents available to tend to children [41].

Conversely, other studies indicate that polygamy can harm a child’s health. In the Igbo group in Nigeria, it has been reported that the relationship between polygamy and higher rates of child mortality may be largely due to the disadvantaged demographic of women who typically end up in polygamous marriages (i.e. poorer women with little or no education) [38]. This may result in women having little to no sexual and reproductive health autonomy, financial decision-making, or education about the importance of seeking professional healthcare if their child becomes ill [38].

There was a significant relationship between a woman’s partner having a secondary level of education and a lower likelihood of children experiencing U5M. Children born to women whose partners had completed secondary education were 27% less likely to experience U5M compared to children born to women whose partners received no formal education. Although our study focused solely on partners’ education, the protective value of education for both men and women is pivotal to mitigating MHRFBs and their associated consequences to child health, such as U5M. Being educated increases knowledge about infant health (and health in general) and increases the uptake of hygiene-positive health behaviours [28]. The literature illustrates that education is a powerful protective factor against engaging in MHRFBs and reports that women with primary or secondary and higher education and partners with primary or secondary levels of education are less likely to engage in MHRFBs in SSA [5, 32] and in Bangladesh [42]. It is well established that educated women are more likely to have knowledge about family planning and HRFBs [5, 32, 42] and are more likely to have greater financial resources and security. In Nigeria, women with at least a secondary education had a lower risk of their infant dying [7]. In Malawi and Uganda, education has been associated with a reduced likelihood of U5M [43]. Other studies in Nigeria have indicated that mortality can be largely prevented by seeking access to appropriate care services, such as family planning and healthcare services for children [44]. However, a lack of education remains a persistent barrier preventing women from seeking and accessing appropriate care services for themselves and their children.

Female children were 16% less likely to die under five years of age compared to male children. Other studies in Nigeria, and more broadly in LMICs, have reported similar findings, noting that male children are physiologically weaker and more prone to illnesses such as weaker lung development compared to female children [33]. Also, in Nigeria, male neonates had a higher chance of dying compared to females [27]. Similar findings have been observed in India and Pakistan: an analysis of 297,509 total births from 2010 to 2018 revealed that male neonates were significantly more likely to die compared to females in the first week after birth [45], and similar findings were echoed in Indonesia [46].

Children who received postnatal care after birth were 41% less likely to experience U5M compared to children who received no postnatal checks after birth. This emphasises the power of preventative postnatal care visits to reduce U5M. Similarly, other studies in Nigeria reported the same finding, with children who received regular postnatal care visits being less likely to experience U5M [33]. Importantly, postnatal care visits are a key component of maternal and child health services and a key intervention to reduce child mortality in SSA [47, 48]. Several studies have noted that the days and months immediately following childbirth may be the highest risk for U5M. Egbon et al. [33] state that the first two months postpartum are the riskiest for U5M in Nigeria. In LMICs, the first 2 years are generally reported to be the most high-risk period for U5M [49]. A study in Nigeria explored using a mobile health intervention (mHealth), which provides health promotion messaging on users’ mobile phones to promote the uptake and utilisation of postnatal care services [50]. The initiative is intended to increase women’s and partners’ knowledge about appropriate maternal-child care during the postpartum period [50]. In this study, almost every participant reported feeling they had increased awareness of postnatal care and the importance of accessing these services for their newborns [50]. This emphasises the potential of technological applications such as mHealth to drive the uptake of maternal-newborn health services to reduce maternal-child morbidity and mortality. Tools such as this would be an important consideration to integrate into future maternal-child health promotion initiatives in Nigeria.

### Implications for Research and Policy

Our analyses generate new insights into the factors influencing U5M in Nigeria and have important implications for policy change to mitigate the high rates of U5M in the region.

Our analyses indicate that maternal experience of MHRFBs is significantly associated with a higher likelihood of their children dying under the age of 5 in Nigeria. In addition, several social determinants of health were also significant predictors of U5M and included women’s age, age at first marriage, educational level, place of residence, type of marriage, work status, household wealth index, sex of household head, religion, number of antenatal care visits, and postnatal check for the newborn. The factors that were significantly associated with U5M in Nigerian children in our study included the maternal experience of MHRFBs, household wealth index, type of marriage, partner’s education level, sex of the child, and whether the child received postnatal care after birth. Notably, children born to women who experienced MHFRB, children born into polygamous marriages, male children, and children who didn’t receive postnatal care after birth had a higher likelihood of dying before age 5. Strong predictors of a reduced likelihood of U5M included being born to a mother who didn’t experience MHRFBs, being born into the richest household wealth index category, being born into a monogamous marriage, being born to a woman whose partner completed secondary education, being a female child, and receiving postnatal care after birth.

Our findings illuminate key areas that require significant focus and intersectoral collaboration by policymakers, private sectors, nonprofit organizations, and healthcare actors to reduce engagement in MHRFBs that pose a serious risk to U5M in Nigeria. We recommend focusing on four main areas to improve gender equity and mitigate the devastating impact of MHRFBs on women and their children in the country. We recommend: (1) to strengthen educational opportunities to reduce attrition rates for girls in school to improve gender equity, (2) to mobilise adaptive educational programs around family planning that are equally tailored to women and their partners to reduce MHRFBs and associated risk of U5M, (3) to integrate mobile tools and use mass media to strengthen health promotion initiatives and public health messaging to reduce U5M, and (4) to improve accessibility to postnatal care services, particularly in rural areas.

Strengthening educational opportunities for girls and ensuring that they stay in school by mitigating attrition rates will be vital to addressing the root causes driving engagement with MHRFBs. It is well established in the literature that providing educational opportunities for girls is vital to increasing knowledge about reproductive health and family planning, promoting greater personal and health autonomy, and securing greater financial and personal independence. Girls who remain in school longer are also less likely to become child brides and have their education halted consequently. Remaining in school reduces girls’ likelihood of experiencing early pregnancy and engaging with MHRFBs that increase the chances of maternal, neonatal and child under five morbidity and mortality. To achieve SDG3, it will be pivotal to focus on promoting the importance of education, particularly for girls. This will require a cultural shift to transform pervasive gender norms and the patriarchal societal expectations of girls and women. The Nigerian government should maintain education at the forefront of all public health initiatives. Protecting girls’ right to education will be a key factor in lowering the rates of child marriage in the country and is a key preventative upstream strategy to mitigate the negative downstream effects of engagement with MHRFBs that are largely influenced by lower education levels.

However, interpreting this study’s result should be implemented cautiously for policy and developmental decisions because the dataset was collected 5-years before these findings.

### Limitations

DHS data is acquired from interviewing participants and runs the risk of recall bias; thus, participants may not accurately remember specific events or dates during the completion of the questionnaire. The variable on under-five survival status was collected based on a verbal report from the mother; this could be a major limitation as the data could be fraught with errors, including memory bias/recall errors. In addition, the respondents’ understanding of the question on child survival could have been affected by subjective interpretation and some cultural/linguistic barriers. Another important limitation of the study to note is that it focuses exclusively on the most recent data from the 2018 Nigerian DHS, thus the interpretation of this study’s result should be implemented with caution for policy and developmental decision due to the year of data collection.

### Strengths

DHS data are nationally representative and have been collected with a standardised process, allowing for efficient analyses across many countries. This study analysed available data from the 2018 Nigerian DHS. Our analysis included data from 10,304 women of reproductive age 15–49 years who met the study criteria. This study contributes a robust analysis and generates novel insights into the little-explored association between MHRFBs and U5M in Nigeria. It provides important recommendations for policy change to reduce engagement with MHRFBs and improve other socioeconomic factors that ultimately contribute to Nigeria’s high U5M rate.

### Conclusion and recommendations

This study concludes that children under five years of age in Nigeria were more likely to die before their fifth birthday if their mothers engaged in MHRFBs. Children also had a higher risk of U5M if they were born into polygamous marriages. The covariate results also show that protective factors that reduce the likelihood of U5M included being born into the richest household wealth index category, having a mother whose partner completed secondary education, being a female child, and receiving postnatal care after birth. Nigeria has one of the highest rates of U5M both in Africa and globally. The dense population of the country, paired with the exorbitant maternal and U5M rates, pose a grave danger to the lives of women and their children in Nigeria. We recommend strengthening educational opportunities for girls and ensuring that they stay in school longer to curb child marriage rates, which are often accompanied by consequences such as the loss of personal and financial autonomy, associated risks to forced marriage such as MHRFBs, and high maternal and child mortality rates. We also recommend creating and mobilising adaptive reproductive health education programs targeted to the unique needs of women and their partners throughout the diverse regions of Nigeria. We recommend applying mobile tools and utilising mass media to strengthen health promotion initiatives and public health messaging to reduce U5M in the country. Finally, we recommend improving accessibility to postnatal care services, particularly in rural areas, in order to promote preventative and early care-seeking and curb U5M rates.

## Data Availability

The datasets utilised in this study can be accessed at https://dhsprogram.com/data/available-datasets.cfm.

## Abbreviations

U5M: Under-five Mortality IPV:Intimate Partner Violence
SSA: Sub-Saharan Africa
HRFBsS: high-risk fertility behaviours, MHRFBs:multiple high-risk fertility behaviours
SIPV: Sexual intimate partner violence
LMICs: low- and middle-income countries
DHS: Demographic and Health Survey
PSU: Primary survey unit
COR: unadjusted model
AOR: Adjusted Model
CI: Confidence Intervals
UNDESA: United Nations Department of Economic and Social Affairs

## References

[1] Amir-ud-Din R, Naz L, Rubi A, Usman M, Ghimire U (2021). Impact of high-risk fertility behaviours on underfive mortality in Asia and Africa: evidence from demographic and health surveys. BMC Pregnancy Childbirth.

[2] Organisation WH. Birth spacing: report from a WHO technical consultation. 2006.

[3] Goodwin MM, Gazmararian JA, Johnson CH, Gilbert BC, Saltzman LE, Group PW (2000). Pregnancy intendedness and physical abuse around the time of pregnancy: findings from the pregnancy risk assessment monitoring system, 1996–1997. Matern Child Health J.

[4] Tessema ZT, Tamirat KS (2020). Determinants of high-risk fertility behavior among reproductive-age women in Ethiopia using the recent Ethiopian Demographic Health Survey: a multilevel analysis. Trop Med Health.

[5] Seidu A-A, Ahinkorah BO, Anjorin SS, Tetteh JK, Hagan JE, Zegeye B, Adu-Gyamfi AB, Yaya S (2023). High-risk fertility behaviours among women in sub-saharan Africa. J Public Health.

[6] Asresie MB, Dagnew GW (2022). Association of maternal high-risk fertility behavior and under-five mortality in Ethiopia: community-based survey. PLoS ONE.

[7] Salawu MM, Afolabi RF, Gbadebo BM, Salawu AT, Fagbamigbe AF, Adebowale AS (2021). Preventable multiple high-risk birth behaviour and infant survival in Nigeria. BMC Pregnancy Childbirth.

[8] UNICEF Data. : Monitoring the situation of children and women [https://data.unicef.org/country/nga/].

[9] Deaths. 2018 [https://www.150.statcan.gc.ca/n1/daily-quotidien/191126/dq191126c-eng.htm]].

[10] Kunnuji M, Eshiet I, Ahinkorah BO, Omogbemi T, Yaya S (2022). Background predictors of time to death in infancy: evidence from a survival analysis of the 2018 Nigeria DHS data. BMC Public Health.

[11] UNICEF. State of the World’s children: celebrating 20 years of the convention on the Rights of the child. Unicef; 2009.

[12] Determinants. and consequences of high fertility: a synopsis of the evidence [https://www.documents1.worldbank.org/curated/en/389381468147851589/pdf/].

[13] WHO. : Maternal health in Nigeria: generating information for action. 2019.

[14] Child Mortality. (under 5 years) [https://www.who.int/news-room/fact-sheets/detail/levels-and-trends-in-child-under-5-mortality-in-2020].

[15] Woldeamanuel BT, Gessese GT, Demie TG, Handebo S, Biratu TD (2023). Women’s education, contraception use, and high-risk fertility behavior: a cross-sectional analysis of the demographic and health survey in Ethiopia. Front Global Women’s Health.

[16] Seidu A-A, Jnr JEH, Budu E, Aboagye RG, Okyere J, Sakyi B, Adu C, Ahinkorah BO (2023). High-risk fertility behaviour and undernutrition among children under-five in sub-saharan Africa: a cross-sectional study. BMJ open.

[17] Demographic N (2019). Health Survey 2013. National Population Commission (NPC)[Nigeria] and ICF International. Abuja, Nigeria, and Rockville.

[18] Corsi DJ, Neuman M, Finlay JE, Subramanian S (2012). Demographic and health surveys: a profile. Int J Epidemiol.

[19] Aliaga A, Ren R. The optimal sample sizes for two-stage cluster sampling in demographic and health surveys. ORC Macro; 2006.

[20] Das M, Tóth CG, Shri N, Singh M, Hossain B (2022). Does sexual intimate Partner Violence (IPV) increase risk of multiple high-risk fertility behaviours in India: evidence from National Family Health Survey 2015–16. BMC Public Health.

[21] Adedini SA, Odimegwu C, Imasiku EN, Ononokpono DN (2015). Ethnic differentials in under-five mortality in Nigeria. Ethn Health.

[22] Alawode OA, Okeke SR, Sah RK, Bolarinwa OA (2022). Prevalence and determinants of intention to use modern contraceptives among grand-multiparous women in sub-saharan Africa. Archives of Public Health.

[23] Olowolafe TA, Adebowale AS, Fagbamigbe AF, Bolarinwa OA, Akinyemi JO (2023). Shifts in age pattern, timing of childbearing and trend in fertility level across six regions of Nigeria: Nigeria demographic and health surveys from 2003–2018. PLoS ONE.

[24] Bolarinwa OA, Afaya A, Ajayi KV, Ojo A, Alawode OA (2022). Prevalence and factors associated with the use of long-acting reversible and permanent contraceptive methods among women who desire no more children in high fertility countries in sub-saharan Africa. BMC Public Health.

[25] Harrell J, Frank E, Harrell FE. Binary logistic regression. *Regression modeling strategies: With applications to linear models, logistic and ordinal regression, and survival analysis* 2015:219–274.

[26] Rana S, Midi H, Sarkar S. Validation and performance analysis of binary logistic regression model. In *Proceedings of the WSEAS International Conference on Environmental, Medicine and Health Sciences*. WSEAS Press; 2010: 23–25.

[27] Ezeh OK, Agho KE, Dibley MJ, Hall J, Page AN (2014). Determinants of neonatal mortality in Nigeria: evidence from the 2008 demographic and health survey. BMC Public Health.

[28] SALAMI KK, DUMBILI E, EZEAH P. Determinants of maternal and child healthcare service sutilisation among recently pregnant mothers in Ubulu-Okiti, Delta State Nigeria. Int J Sociol Family 2013:115–27.

[29] Brown W, Ahmed S, Roche N, Sonneveldt E, Darmstadt GL. Impact of family planning programs in reducing high-risk births due to younger and older maternal age, short birth intervals, and high parity. Seminars in perinatology. Elsevier; 2015: 338–44. 10.1053/j.semperi.2015.06.006 26169538

[30] Brown W, Ahmed S, Roche N, Sonneveldt E, Darmstadt GL (2015). Impact of family planning programs in reducing high-risk births due to younger and older maternal age, short birth intervals, and high parity. Semin Perinatol.

[31] Gebrehiwot SW, Abera G, Tesfay K, Tilahun W (2019). Short birth interval and associated factors among women of child bearing age in northern Ethiopia, 2016. BMC Womens Health.

[32] Tamirat KS, Tesema GA, Tessema ZT (2021). Determinants of maternal high-risk fertility behaviors and its correlation with child stunting and anemia in the East Africa region: a pooled analysis of nine east african countries. PLoS ONE.

[33] Egbon OA, Bogoni MA, Babalola BT, Louzada F (2022). Under age five children survival times in Nigeria: a bayesian spatial modeling approach. BMC Public Health.

[34] Dougherty L, Gilroy K, Olayemi A, Ogesanmola O, Ogaga F, Nweze C, Banerjee J, Oduenyi C, Pacqué M (2020). Understanding factors influencing care seeking for sick children in Ebonyi and Kogi States, Nigeria. BMC Public Health.

[35] Nnebue C, Ebenebe U, Uadogu P, Onah S, Onyeonoro U, Ifeadike C, Onwasigwe C (2012). Availability of resources for provision of child health services at the primary health care level in Nnewi, Nigeria. Orient J Med.

[36] Statistics NBo. : Poverty and inequality in Nigeria. 2020.

[37] Uneke CJ, Ndukwe CD, Ezeoha AA, Urochukwu HC, Ezeonu CT (2014). Improving maternal and child healthcare programme using community-participatory interventions in Ebonyi State Nigeria. Int J Health Policy Manage.

[38] Arthi V, Fenske J (2018). Polygamy and child mortality: historical and modern evidence from Nigeria’s Igbo. Rev Econ Househ.

[39] Weekes S, Bangura P, Sesay J. Sierra Leone 2015 Population and Housing Census. 2017.

[40] Ndiaye K, Portillo E, Ouedraogo D, Mobley A, Babalola S (2018). High-risk advanced maternal age and high parity pregnancy: tackling a neglected need through Formative Research and Action. Glob Health Sci Pract.

[41] Ukwuani FA, Cornwell GT, Suchindran CM (2002). Polygyny and child survival in Nigeria: age-dependent effects. J Popul Res.

[42] Howlader MH, Roshid HO, Kundu S, Halder HR, Chanda SK, Rahman MA (2022). Determinants associated with high-risk fertility behaviours among reproductive aged women in Bangladesh: a cross-sectional study. Reproductive Health.

[43] Andriano L, Monden CW (2019). The causal effect of maternal education on child mortality: evidence from a quasi-experiment in Malawi and Uganda. Demography.

[44] Olaitan T, Okafor IP, Onajole AT, Abosede OA (2017). Ending preventable maternal and child deaths in western Nigeria: do women sutilise the life lines?. PLoS ONE.

[45] Aghai ZH, Goudar SS, Patel A, Saleem S, Dhaded SM, Kavi A, Lalakia P, Naqvi F, Hibberd PL, McClure EM (2020). Gender variations in neonatal and early infant mortality in India and Pakistan: a secondary analysis from the Global Network maternal Newborn Health Registry. Reproductive Health.

[46] Titaley CR, Dibley MJ, Agho K, Roberts CL, Hall J (2008). Determinants of neonatal mortality in Indonesia. BMC Public Health.

[47] Wudineh KG, Nigusie AA, Gesese SS, Tesu AA, Beyene FY (2018). Postnatal care service sutilisation and associated factors among women who gave birth in Debretabour town, North West Ethiopia: a community-based cross-sectional study. BMC Pregnancy Childbirth.

[48] Tessema ZT, Yazachew L, Tesema GA, Teshale AB (2020). Determinants of postnatal care sutilisation in sub-saharan Africa: a meta and multilevel analysis of data from 36 sub-saharan countries. Ital J Pediatr.

[49] Karlsson O, Kim R, Hasman A, Subramanian S (2022). Age distribution of all-cause mortality among children younger than 5 years in low-and middle-income countries. JAMA Netw Open.

[50] Olajubu AO, Fajemilehin BR, Olajubu TO (2022). Mothers’ experiences with mHealth intervention for postnatal care utilisation in Nigeria: a qualitative study. BMC Pregnancy Childbirth.

